# Outcomes of adalimumab biosimilar nonmedical switches in children and young adults with inflammatory bowel disease

**DOI:** 10.1002/jpr3.70212

**Published:** 2026-07-05

**Authors:** Daniel Himelstein, Megan McNicol, Mahmoud Abdel‐Rasoul, Brendan M. Boyle, Hilary K. Michel, Ross Maltz

**Affiliations:** ^1^ Division of Pediatric Gastroenterology, Hepatology and Nutrition Nationwide Children's Hospital Columbus Ohio USA; ^2^ Department of Pharmacy Nationwide Children's Hospital Columbus Ohio USA; ^3^ Department of Biomedical Informatics, Center for Biostatistics The Ohio State University Columbus Ohio USA; ^4^ Department of Pediatrics The Ohio State Wexner Medical Center Columbus Ohio USA

**Keywords:** anti‐TNF medications, Crohn's disease, pediatrics, ulcerative colitis

## Abstract

**Objectives:**

Adalimumab biosimilars are as safe and effective compared to the originator. Adult patients with inflammatory bowel disease (IBD) who switched to a biosimilar have comparable outcomes, but pediatric data are limited. This study evaluates clinical outcomes of children and young adults with IBD following a nonmedical, insurance‐driven switch from the adalimumab originator to a biosimilar.

**Methods:**

A single‐center retrospective chart review was conducted among pediatric and young adult patients with IBD who switched from the adalimumab originator to a biosimilar between May 2023 and July 2024. Demographics, Physician Global Assessment (PGA), laboratory values, adalimumab levels, and antibodies were collected preswitch and up to 6 months postswitch. Adalimumab biosimilar continuation was assessed 6 months postswitch. McNemar's exact test, linear mixed effect models, and paired *t*‐tests compared variables preswitch and postswitch.

**Results:**

Fifty patients switched to a biosimilar. Forty‐two patients had PGAs pre and postswitch, and among them, 86% (36/42) of patients demonstrated stable or improved PGAs postwitch. Seventy‐six percent (38/50) of patients continued on the biosimilar for at least 6 months postswitch. Of the 12 patients who discontinued adalimumab biosimilar, 42% (5/12) discontinuation was not related to the switch, while 58% (7/12) were due to the switch. Laboratory values remained stable pre and postswitch, although adalimumab levels decreased postswitch (18–15 µg/mL; *p* = 0.007).

**Conclusions:**

Switching from the adalimumab originator to a biosimilar resulted in comparable outcomes in children and young adults with IBD based on PGA, laboratory markers, and continuation of the medication.

## Abstract (highlights)

**What is Known:**

- Adalimumab is approved for adults and children with Crohn's disease and ulcerative colitis by the United States Food and Drug Administration.
- Adalimumab biosimilars were first available in the United States in 2023.
- There is similar safety and efficacy between adalimumab originator and biosimilars.

**What is New:**

- Pediatric patients who underwent a one‐time nonmedical switch from adalimumab originator to a biosimilar had similar clinical outcomes based on Physician Global Assessment.
- Laboratory and biochemical markers were similar following a switch to an adalimumab biosimilar.
- Most patients remained on an adalimumab biosimilar 6 months after a nonmedical switch.

## 1 INTRODUCTION

Infliximab and adalimumab are anti‐tumor necrosis factor (anti‐TNF) biologic therapies used as first‐line treatments in children and young adults with inflammatory bowel disease (IBD).^1^ Although highly effective, these medications are major contributors to rising healthcare costs in IBD.^2^, ^3^, ^4^ To mitigate costs, biosimilars—biologic medications highly similar to their originators—have been introduced into clinical practice.^5^

Infliximab biosimilars, which became available in the United States in 2016, have comparable safety and efficacy in adult and pediatric patients with IBD.^6^, ^7^, ^8^, ^9^, ^10^ Their lower cost has led insurance payors to mandate nonmedical switches from originator products to biosimilars.^11^ Importantly, nonmedical switches of infliximab have been shown to be safe and effective in both adults and pediatric IBD populations.^12^, ^13^, ^14^

While infliximab biosimilars have been available in the United States since 2016, adalimumab biosimilars were first available in the United States in 2023. Several studies have shown similar safety and efficacy in adult and pediatric patients using adalimumab biosimilars^15^, ^16^, ^17^, ^18^, ^19^, ^20^, ^21^; however, most of the literature focuses on biosimilar use in adalimumab‐naïve patients. There is a paucity of studies looking at the effects of nonmedical switches of adalimumab in children and young adults with IBD in the United States.

The primary aim of this study was to assess maintained or improved clinical disease status, measured by Physician Global Assessment (PGA), after a nonmedical switch from the adalimumab originator to a biosimilar in children and young adults with IBD. Secondary outcomes were to assess whether nonmedical switches affected laboratory markers and biosimilar continuation at 6 months postswitch.

## 2 METHODS

### 2.1 Ethics statement

Ethical approval for the study was obtained from Nationwide Children's Hospital (NCH) Institutional Review Board (STUDY00002869). Informed consent was waived secondary to the retrospective nature of the study.

### 2.2 Study design

This single‐center retrospective review was performed at NCH in Columbus, Ohio. All children and young adults (18–25 years old) with IBD who were prescribed the adalimumab originator and subsequently underwent a nonmedical, insurance‐mandated switch to an adalimumab biosimilar between May 2023 and July 2024 were eligible for inclusion. Patients were excluded if they were not receiving the originator prior to the switch or if they did not maintain care at NCH for at least 6 months following the switch. Data included sex, race, Paris classification of IBD,^22^ type of insurance, and patient age at the time of the nonmedical switch.

The primary objective was to assess clinical status after the switch using the PGA. The PGA is a validated tool for evaluating disease activity in patients with IBD, incorporating symptoms, physical examination findings, laboratory values, and functional status. By integrating these factors, clinicians can generate an overall assessment of disease severity. Disease activity is categorized as follows: quiescent disease (minimal or no symptoms, normal laboratory values, and no active fistula or weight loss); mild disease (intermittent or mild symptoms occurring several times per week, with persistent laboratory abnormalities but no fistula or weight loss); moderate disease (more pronounced symptoms, significant laboratory abnormalities, and possible fistula, abdominal tenderness, or unexplained weight loss); and severe disease (marked symptoms, substantial functional impairment, markedly abnormal laboratory values, and the presence of fistula, abdominal mass, or toxic appearance).^23^, ^24^ The primary objective was to assess clinical status after the switch via the PGA. PGA scores were standardized across all providers at our institution, and were obtained from the office visit closest to the switch date (up to 1 year prior) and from a follow‐up visit within 6 months postswitch. PGA was used in lieu of other comprehensive clinical scoring tools, such as the Pediatric Crohn Disease Activity Index (PCDAI) and the Pediatric Ulcerative Colitis Activity Index (PUCAI), as it is more readily available for use in retrospective studies. Further, several studies have demonstrated moderate correlation between PGA and PCDAI and moderate‐to‐strong correlation between PGA and PUCAI.^23^, ^24^

Secondary outcomes included laboratory values, biochemical markers, and biosimilar durability. Laboratory values, including hemoglobin (Hgb), albumin, erythrocyte sedimentation rate (ESR), C‐reactive protein (CRP), adalimumab true trough level, and antidrug antibodies (ADA) via drug‐tolerant assay (ARUP Laboratories) were collected up to 12 months prior to the switch and up to 6 months postswitch. If the patient transitioned back to the originator or to a different therapeutic class, postswitch laboratory and PGA data were only included if obtained while the patient was still receiving the biosimilar. Data collected after discontinuation of the biosimilar were excluded. At 6 months postswitch, patients were assessed to determine whether they remained on the adalimumab biosimilar. For those who discontinued, the reason for therapy change was recorded. Office visit notes, telephone encounters, and patient messages were manually reviewed to record potential side effects or intolerances following a switch to the biosimilar medication.

### 2.3 Statistical analysis

Results were reported as means with 95% confidence intervals. Several different statistical methods were used to assess differences in variables before and after nonmedical switches at the specified time points. Wilcoxon signed‐rank test was utilized for comparing PGA values, whereas McNemar's exact test was utilized for comparing ADA. Linear mixed‐effect models with random intercepts were employed for comparing lab values. Paired *t*‐tests were used to compare dose and interval. All data were analyzed using SAS version 9.4 (SAS Institute), with a *p*‐value of <0.05 considered statistically significant.

## 3 RESULTS

Fifty patients met inclusion criteria and underwent a nonmedical switch from the adalimumab originator to a biosimilar. The cohort was predominantly male (58%), white (90%), diagnosed with Crohn's disease (CD) (88%), and had commercial insurance (100%) (Table 1). Eight percent were on concomitant immunomodulator or steroids at the time of switch. Although multiple adalimumab biosimilars have been approved and utilized, the majority of patients (92%) in this study were switched to adalimumab‐adaz due to payor formularies.

**Table 1:** Patient demographics.

**Patient demographics (*n*** = **50)**	
Sex	*n* (%)
Male	29 (58)
Ethnicity	*n* (%)
Nonhispanic	49 (98)
Disease	*n* (%)
Crohn's disease	44 (88)
UC/IBD‐unclassified	6 (12)
Biologic use prior to adalimumab	*n* (%)
Biologic naïve	44 (88)
Prior infliximab use	6 (12)
Prior other biologic/small molecule	0 (0)
Characteristics at time of switch	Median years (range)
Adalimumab originator treatment duration	3.2 (1.1–5.3)
Age at switch	17.3 (11.5–23.6)
Postswitch medication	*n* (%)
Adalimumab‐adaz	46 (92)
Adalimumab‐atto	2 (4)
Adalimumab‐bwwd	1 (2)
Adalimumab‐aacf	1 (2)
Dose and frequency at switch	*n* (%)
20 mg	
Every 7 days	0 (0)
Every 10 days	0 (0)
Every 14 days	5 (10)
40 mg	
Every 7 days	11 (22)
Every 10 days	1 (2)
Every 14 days	29 (58)
80 mg	
Every 7 days	1 (2)
Every 10 days	0 (0)
Every 14 days	3 (6)
Insurance type	*n* (%)
Commercial/private	50 (100)
Concommitant therapy at switch	*n* (%)
6‐MP/azathioprine	0 (0)
Methotrexate	3 (6)
Steroids	1 (2)
None	46 (92)

Abbreviations: 6‐MP, 6‐mercaptopurine; IBD, inflammatory bowel disease; UC, ulcerative colitis.

Forty‐two patients had a PGA recorded pre and postswitch, whereas eight patients had a preswitch PGA but no postswitch PGA (Table 2). Of those, 86% (36/42) were in clinical remission after switching to the biosimilar, including 74% (31/42) who remained in remisson and 12% (5/42) who improved to remission. Disease activity worsened in 14% (6/42) of patients. Seven percent (3/42) worsened from quiescent to mild disease, and 2% (1/42) worsened from quiescent to moderate disease. The Wolcoxon signed‐rank test showed no significant change in disease activity when comparing paired PGA measurements before and after switching to the biosimilar (*p* = 0.79). When comparing laboratory values using linear mixed effects models before and after the nonmedical switch, there were no statistically significant differences observed in albumin, Hgb, CRP, or ESR (Table 3). Although adalimumab trough levels decreased significantly from 18.1 µg/mL (95% confidence intervaI: 16.2, 20.1) preswitch to 15.0 µg/mL (13.0, 17.0) postswitch (*p* = 0.007), levels remained therapeutic (>10 µg/mL).^25^ Notably, among patients with measured antibodies at both periods (pre and post), 4.8% (2/42) patients had ADA preswitch, whereas 14.3% (6/42) had ADA postswitch (*p* = 0.16).

**Table 2:** Physician global assessment pre and postswitch.

**PGA postswitch, *n*** *		**PGA preswitch, *n***		
	**Quiescent**	**Mild**	**Moderate**	**Total**
Quiescent	31	5	0	36
Mild	3	0	0	3
Moderate	1	2	0	3
Total	35	7	0	42

Abbreviation: PGA, Physician Global Assessment.

* Eight patients had missing values postswitch.

**Table 3:** Laboratory values pre and postswitch.

	**Preswitch**	**Postswitch**	***p*‐value**
Lab value mean (95% CI), (*N*)			
Albumin (g/dL)^a^	4.4 (4.4, 4.5), (48)	4.5 (4.4, 4.6), (44)	0.39
C‐reactive protein (mg/dL)^a^	0.5 (0.4, 0.6), (48)	0.5 (0.4, 0.6), (44)	0.63
HgB (g/dL)^a^	13.9 (13.5, 14.3), (48)	14.0 (13.7, 14.4), (46)	0.21
Erythrocyte sedimentation rate (mm/h)^a^	9.5 (7.2, 11.8), (47)	8.1 (5.8, 10.5), (45)	0.20
Drug levels			
Adalimumab level (μg/mL), mean (95% CI), (*N*)^a^	18.1 (16.2, 20.1), (42)	15.0 (13.0, 17.0), (42)	0.007
Detectable anti‐drug antibodies (ng/mL), *n* (%)^b^	2/42 (4.8)	6/42 (14.3)	0.157
Months postswitch true trough obtained (median, IQR)		8.6 (7.4, 9.9)	

Abbreviations: CI, confidence interval; Hgb, hemoglobin; IQR, interquartile range; N, number.

^a^ Mean (95% CI) and *p*‐value based on linear mixed effect models with random intercepts.

^b^ *p*‐value based on McNemar's test.

Of the 50 patients who underwent a nonmedical switch, 76% (38/50) remained on the adalimumab biosimilar for at least 6 months. The remaining 24% (12/50) of patients discontinued therapy for various reasons outlined in Table 4. Among them, 14% (7/50) of patients switched back to the originator, and 10% (5/50) changed to a different medication, either an alternative anti‐TNF (infliximab) or therapy with a different mechanism of action. Of the 12 patients who discontinued the adalimumab biosimilar, 42% (5/12) discontinued due to reasons unrelated to the switch (one patient insurance change, one patient anxiety with injections, and three patients had persistent disease activity), while 58% (7/12) discontinued due to reasons attributed to the switch (three patients developed adverse reactions, three patients had disease worsening postswitch, and one patient had a decrease in drug level and developed antibodies). Additionally, three patients underwent dose adjustments during the study period. Of these, one required an increased dosing frequency due to a clinical change; the patient remained on the biosimilar at 6 months postswitch and continued in remission at that time. The remaining dose adjustments were due to insurance requirements or increased weight.

**Table 4:** Switch‐back patient cases.

**Category**	**Patient**	**Description**
Disease worsening before switching	1	Reported loose stools and urgency at the time of switch with elevated fecal calprotectin at 1829. Symptoms continued and repeat fecal calprotectin was 2727. Changed medication to upadacitinib with normalization of fecal calprotectin.
	2	Reported partial response to adalimumab originator when switched to the biosimilar. Eventually was switched to infliximab after 3.5 months of biosimilar. No repeat labs obtained or esophagogastroduodenoscopy/colonoscopy performed at time of switches.
	3	Active inflammation prior to switch with fecal calprotectin 1676, with continued inflammation and oral involvement following switch. Subsequently switched to ustekinumab.
Unrelated to switching	4	Switch back due to insurance change, not due to symptoms.
	5	Reported no symptoms, but had anxiety related to injections, switched to infliximab after 3 months of biosimilar.
Adverse reaction attributed to switching	6	Reported no change in Crohn's disease symptoms, but developed injection site pain and hives. Switched back and had resolution of symptoms.
	7	Reported hair loss, joint pain, and acne following three doses of biosimilar despite level of 12.5. Switched back with normal esophagogastroduodenoscopy/colonoscopy after.
	8	Reported fatigue, headaches, loose stools, hair loss, and swollen lymph nodes with 3 months of biosimilar. Had a fecal calprotectin of <27 before switch to biosimilar, while on biosimilar, and following switch back to originator.
Clinically significant decreased drug level after switching	9	Reported no symptoms, but adalimumab level decreased from 27.9 to 4.2, also developed antibodies to 209. Switched back due to patient preference with a higher dose and level remained low. Dosing adjusted again and now has adequate level.
Disease worsening after switching	10	Reported swollen inguinal lymph nodes following switch, with increase in fecal calprotectin from 208 to 976 on biosimilar. Switched back to originator and fecal calprotectin decreased to 575, but increased back to 1019.
	11	Reported increased abdominal pain, frequency of stools, looser stools, weight loss, and fatigue. Switched back after 2 months and symptoms resolved.
	12	Reported some abdominal pain and blood in stools, developed high adalimumab antibodies so switched to infliximab.

## 4 DISCUSSION

To our knowledge, this is the first US‐based study examining the impact of nonmedical switching from the adalimumab originator to a biosimilar in children and young adults with IBD. While there is limited data on adalimumab switching in pediatric IBD, previous studies involving nonmedical switches with infliximab biosimilars have demonstrated comparable safety and efficacy in this population.^12^, ^13^, ^14^ In adult studies, switching from the adalimumab originator to a biosimilar have shown similar durability, clinical outcomes, and biochemical markers.^26^, ^27^, ^28^, ^29^

The primary outcome measure used in this study to assess clinical change following a switch to an adalimumab biosimilar was PGA. In our cohort, 42 patients had a PGA documented both pre and postswitch, and 86% (36/42) had either stable or improved disease activity postswitch. No patients developed severe disease activity. This aligns with adult IBD studies, where 82% of patients maintained clinical remission 6 months after switching.^30^

Our secondary outcomes of laboratory and biochemical markers were also supportive of clinical stability following a switch. Albumin, ESR, CRP, and Hgb were all unchanged following a switch. We did note a statistically significant decrease in drug level from 18.1 to 15.0 μg/mL (*p* = 0.007). For patients with perianal disease this decrease could be significant given higher troughs have been shown to increase fistula healing and fistula closing.^31^ However, we do not suspect that this difference is clinically meaningful for those without perianal disease, as the adalimumab levels pre and postswitch were still therapeutic >10 (μg/mL).^25^ The presence of ADA did increase from 4.8% (2/42) preswitch to 14.3% (6/42) postswitch. While not statistically significant, this may also be clinically meaningful.

Biosimilar continuation was also assessed as part of our secondary outcome measures. Seventy‐six percent (38/50) of patients remained on the biosimilar 6 months after the insurance‐driven switch. This is slightly lower than a European cohort, where 93% of pediatric patients remained on the adalimumab biosimilar at 6 months.^20^ That study also found a lower risk of treatment failure in patients who switched from the originator compared to those who were adalimumab‐naïve and started on a biosimilar outright. The decline in patient retention on the adalimumab biosimilar may not be specifically due to the biosimilar itself; the adalimumab originator demonstrated continuation rates of 98% at 14 weeks, 90% at 1 year, and 77% at 3 years in patients with CD, and 83% at 14 weeks, 46% at 1 year, and 37% at 3 years in patients with ulcerative colitis/IBD‐unspecified (UC/IBD‐U).^32^

In this cohort, 6% (3/50) patients had clinical worsening attributed to failure to the biosimilar, resulting in discontinuation of the medication prior to 6 months. Among these three patients, two had corresponding changes in biochemical markers, while one had symptom recurrence without objective data. This raises the possibility of a nocebo effect, a well‐described phenomenon in pharmacology in which a patient's expectations create a nonphysiologic negative effect, specifically during biosimilar transitions.^33^, ^34^, ^35^

Our study population consisted predominantly of patients with CD. In review of the patients with UC or IBD‐U, 33% (2/6) patients had worsened PGAs following the switch to the adalimumab biosimilar, whereas 33% (2/6) others were no longer on the biosimilar at 6 months following a switch. The laboratory values and drug levels for the UC and IBD‐U population otherwise did not differ relative to the CD population. We suspect these findings are secondary to the small population of patients with UC and IBD‐U in our sample, but future studies are needed in these populations.

In this pediatric and young adult cohort, 6% (3/50) of patients developed side effects attributed to the adalimumab biosimilar, including injection site reactions, joint pain, hair loss, and lymphadenopathy—all of which are known adverse effects of adalimumab products.^16^, ^36^, ^37^, ^38^, ^39^, ^40^ These patients all had switched to adalimumab‐adaz, which raises the question of whether the adverse effects are secondary from that specific product. Adalimumab‐adaz is 1 of 10 products on the US market, and thus these specific adverse effects cannot be generalized to all adalimumab biosimilars. In addition, each biosimilar product has differing features of the delivery device, including formulation, presence of citrate, and needle gauge, which could contribute to tolerability of a medication.

There are several limitations of this study that are important to note. This study was a single‐center design and may not reflect broader prescribing patterns, which are influenced by institutional policies, insurance coverage, and geographic location. Additionally, the recent availability of adalimumab biosimilars in the United States constrained both sample size and follow‐up duration. As a result, several demographic features of the patient population were skewed, leading to risk of selection bias, including disease process (predominantly CD), ethnicity (predominantly white), and insurance type (all commercial). The study was also insufficiently powered to detect differences in immunogenicity, subgroup outcomes, and uncommon adverse events, potentially increasing the risk of type II error.

The retrospective nature of this study also lends itself to several limitations. Preswitch laboratory values were collected up to 1 year before the switch, which was necessary as laboratory values at the exact time of switching were not feasibly obtained retrospectively. However, this introduces the possibility that preswitch laboratory values are not reflective of the patient's clinical status at the time of switch and may reflect natural disease fluctuations rather than effects attributable to the biosimilar. For several patients, laboratory data or PGA scores were missing. When available, patient data were included in the analysis with the acknowledgment that this missing data is a limitation. Including only postswitch data from patients who remained on therapy may introduce survivorship bias, potentially overestimating treatment stability.

The current study exclusively evaluates clinical and pharmacokinetic outcomes related to adalimumab biosimilars and does not measure economic outcomes. Additional studies pairing this data with cost analysis will help better understand the impact of biosimilars on payers and consumers alike.

## 5 CONCLUSION

In summary, clinical, laboratory, and pharmacokinetic outcomes were largely maintained over 6 months following a nonmedical switch to adalimumab biosimilars. While short‐term medication continuation was demonstrated, larger and longer‐term studies are needed to better understand biosimilar use in children—especially as the economic landscape continues to evolve.

## CONFLICT OF INTEREST STATEMENT

The authors declare no conflicts of interest.
